# Separation of influenza virus‐like particles from baculovirus by polymer‐grafted anion exchanger

**DOI:** 10.1002/jssc.201901215

**Published:** 2020-04-30

**Authors:** Katrin Reiter, Patricia Pereira Aguilar, Dominik Grammelhofer, Judith Joseph, Petra Steppert, Alois Jungbauer

**Affiliations:** ^1^ Austrian Centre of Industrial Biotechnology Vienna Austria; ^2^ Department of Biotechnology University of Natural Resources and Life Sciences Vienna Austria

**Keywords:** anion exchange chromatography, downstream processing, HIV‐1 gag, insect cells, vaccines

## Abstract

The baculovirus expression vector system is a very powerful tool to produce virus‐like particles and gene‐therapy vectors, but the removal of coexpressed baculovirus has been a major barrier for wider industrial use. We used chimeric human immunodeficiency virus‐1 (HIV‐1) gag influenza‐hemagglutin virus‐like particles produced in *Tnms*42 insect cells using the baculovirus insect cell expression vector system as model virus‐like particles. A fast and simple purification method for these virus‐like particles with direct capture and purification within one chromatography step was developed. The insect cell culture supernatant was treated with endonuclease and filtered, before it was directly loaded onto a polymer‐grafted anion exchanger and eluted by a linear salt gradient. A 4.3 log clearance of baculovirus from virus‐like particles was achieved. The absence of the baculovirus capsid protein (vp39) in the product fraction was additionally shown by high performance liquid chromatography‐mass spectrometry. When considering a vaccination dose of 10^9^ particles, 4200 doses can be purified per L pretreated supernatant, meeting the requirements for vaccines with <10 ng double‐stranded DNA per dose and 3.4 µg protein per dose in a single step. The process is simple with a very low number of handling steps and has the characteristics to become a platform for purification of these types of virus‐like particles.

**Article related abbreviations**
AIEX: anion exchange chromatography
BEVS: baculovirus expression vector system
BSA: bovine serum albumin
CV: column volume
dsDNA: double‐stranded deoxyribonucleic acid
FT: flow‐through
Gp64: glycoprotein 64
HA H1: hemagglutinin 1
HEPES: 4‐(2‐hydroxyethyl)‐1‐piperazineethanesulfonic acid
HIV‐1: human immunodeficiency virus‐1
LDS: lithium dodecyl sulfate
LS: light scattering
MALS: multiangle light scattering
MOI: multiplicity of infection
NaCl: sodium chloride
NaOH: sodium hydroxide
NTA: nanoparticle tracking analysis
Sf9: *Spodoptera frugiperda* 9
TCID50: tissue culture infective dose 50
TMAE: trimethylammoniumethyl
VLP: virus‐like particle

## 1 INTRODUCTION

The insect‐cell baculovirus expression vector system (BEVS) has been widely used for industrial manufacturing of vaccines [1, 2] and gene‐therapy vectors [3]. The major challenge for effective downstream processing of virus‐like particles (VLPs) produced using BEVS is the coexpression of baculovirus and other extracellular vesicles (EVs) alongside VLPs [4, 5]. VLPs based on the human immunodeficiency virus‐1 (HIV‐1) gag construct are spherical nanoparticles with a diameter between 100 and 200 nm, surrounded by a lipid envelope [6, 7, 8, 9]. Baculoviruses are rod shaped, enveloped double‐stranded DNA (dsDNA) viruses with a particle size of 30–70 nm in diameter and 200–400 nm in length [10, 11]. During budding from the host cell, baculovirus nucleocapsids obtain a host cell‐derived envelope which is enriched with the baculovirus major envelope glycoprotein gp64 [12, 13]. The nucleocapsid core is composed mainly by the major capsid protein vp39, which encapsulates the viral genome and is used as specific marker for the presence of baculovirus [14]. Separation and discrimination of VLPs and baculovirus is challenging due to their overlap in size and buoyant densities [15]. Therefore, efficient separation of these particles cannot be performed by density gradient centrifugation or size exclusion chromatography [16, 17, 18]. Additionally, these strategies often do not fulfill the purity specifications of VLP/vaccine preparations for human application. VLPs and baculovirus show similar composition of membrane proteins as both particles bud directly from the plasma membrane of the host cell. It has been shown that enveloped VLPs based on the HIV‐1 gag construct produced in BEVS display viral or cellular membrane proteins on their surface and can also carry the baculovirus encoded major envelope glycoprotein gp64 [19, 20, 21]. This complicates the purification of these types of particles even more and, thus, detailed characterization of samples is only possible by using a combination of several analytical methods. The first VLP‐based vaccine produced in the BEVS was a vaccine against cervical cancer [22], approved in 2009 [23], but this is a protein particle and therefore substantially different to baculovirus. Similar, the recombinant hemagglutinin(HA)‐based trivalent influenza vaccine FluBlok approved by food drug administration (FDA) in 2013 [24], is protein based and thus very different to baculovirus. We used HIV‐1 gag influenza H1 VLPs expressed in *Tnms*42 insect cells as model system. These chimeric VLPs are enveloped VLPs composed of the HIV‐1 gag capsid protein and the influenza A virus derived HA H1. H1 is one of the subtypes of the major influenza surface glycoprotein HA, to which antibodies are able to bind resulting in the agglutination of virus particles and consequently, enabling virus neutralization [25]. Therefore, HA is immune dominant and the main antigen in the VLPs [26], besides the HIV‐1 gag capsid protein. We developed a downstream process based on anion‐exchange chromatography (AIEX) for capture and purification of HIV‐1 gag H1 VLPs using Fractogel^®^‐Trimethylammoniumethy (TMAE) as stationary phase. Fractogel^®^‐TMAE is a polymer‐grafted ion exchange medium, consisting of synthetic methacrylate porous beads with long linear polymer chains (“tentacles”), carrying the functional groups, TMAE. These so‐called “tentacles” are covalently attached to the hydroxyl groups of the matrix, increasing the surface area and number of ligands available for binding. Fractogel^®^‐TMAE shows a particle size of 40–90 µm and a pore size of approximately 80 nm [27]. This work presents the establishment of an AIEX method, based on polymer‐grafted media, to successfully separate HIV‐1 gag H1 VLPs from coexpressed baculovirus produced using *Tnms*42 insect cells. The use of Fractogel^®^‐TMAE allowed the capture and purification of enveloped VLPs including separation of VLPs and baculovirus and reduction of host cell proteins and DNA in a single step. The developed method is suitable for fast and simple downstream processing of enveloped VLPs produced using insect‐cell BEVS and allows the direct loading of endonuclease‐treated cell culture supernatant onto the column.

## 2 MATERIALS AND METHODS

### 2.1 Chemicals and standards

All chemicals were of analytical grade, if not otherwise stated. Sodium chloride (NaCl), sodium hydroxide (NaOH), SDS, 2‐(*N*‐moprholino)ethanesulfate acid (MES), Tween‐20, sulfuric acid (95–97%, H_2_SO_4_), uranyl acetate were purchased from Merck (Darmstadt, Germany).

4‐(2‐Hydroxyethyl)‐1‐piperazineethanesulfonic acid (HEPES) (≥99.5%), 2‐propanol, bovine serum albumin (BSA) (≥99.5%), 1,4‐dithiotreitol (DTT), anti‐mouse IgG (γ‐chain specific)‐alkaline phosphatase antibody (#3438), BCIP^®^/NBT solution, triton X‐100, glutaraldehyde solution (grade I), acetonitrile (MS grade), formic acid (98–100%), and iodacetamide (≥99%) were purchased from Sigma Aldrich (St. Louis, MO, USA). Anti‐rabbit IgG (H+L) secondary antibody (**#**31460), anti‐mouse IgG (H+L) superclonal secondary antibody (**#**A28177) were purchased from Thermo Fisher (Waltham, MA, USA). SeeBlue^®^ plus 2 prestained protein standard and 4× LDS sample buffer were purchased from Invitrogen (Carlsbad, CA, USA). C‐LEcta Denarase^®^ was purchased from VWR (Radnor, PA, USA), HIV‐1 p24 antibody (ab9071) and ACV5 (ab49581) from Abcam (Cambridge, UK), influenza A virus H1N1 HA (GTX127357) from GeneTex (Irvine, CA, USA) and trypsin from Promega (Madison, WI, USA).

### 2.2 Production of virus‐like particles

#### 2.2.1 Preculture

For the cultivation of HIV‐1 gag H1 VLPs, *Tnms*42 cells were kept in exponential growth phase at 27°C in shaker flasks at 100 rpm. The cells were grown in serum‐free medium (Hyclone SFM4Insect, GE Healthcare, Uppsala, Sweden) supplemented with 0.1% Kolliphor P188 (Sigma Aldrich). Viable cell counts were determined by trypan blue exclusion using an automated cell counter (TC20 Bio‐Rad Laboratories, Hercules, CA, USA). For each experiment, cells were taken from adherent culture, transferred to suspension with a starting cell density of 0.5 × 10^6^ cells/mL, and grown to desired cell numbers. All precultures with *Tnms*42 cells were supplemented with heparin sodium (1:1000, Sigma Aldrich) to avoid cell clumping.

#### 2.2.2 Benchtop bioreactor cultivations

Production was performed in a 10 L single use bioreactor (BioBLU 10c, Eppendorf) equipped with one pitched‐blade impeller (3 blades; 45°). The temperature was set to 27°C and the pH maintained at 6.4 ± 0.05 using 25% v/v phosphoric acid and 7.5% w/v sodium bicarbonate. The dissolved oxygen level was maintained at 30%. Cells were inoculated at a cell density of 1 × 10^6^ cells/mL and cultivated in the bioreactor for 1 day prior to infection. Cell count in the bioreactor was determined, and the vessel was infected with the respective amount of baculovirus (MOI = 5) and diluted back to 1 × 10^6^ cells/mL.

#### 2.2.3 Clarification

Cell culture supernatant was harvested after 66 h with a viability of 54% and clarified by low‐speed centrifugation at 200 g for 30 min and 0.01% NaN_3_ was added to inhibit microbial growth. Culture supernatant was either stored in the cold room at 4°C or was frozen at −80°C for long time storage.

### 2.3 Chromatographic workstation

All chromatographic experiments were performed with an Äkta Pure 25 M2, equipped with a sample pump S9 and a fraction collector F9‐C (GE Healthcare). Unicorn software 6.4.1 was used for data collection and analysis. During the purification runs, UV absorbances (280, 260, and 214 nm) and conductivity were monitored simultaneously.

### 2.4 Capture and purification of virus‐like particles using Fractogel^®^‐TMAE

*Tnms*42 supernatant containing HIV‐1 gag H1 VLPs and baculovirus was incubated with c‐LEcta Denarase^®^ (purity > 99%, VWR) at a final concentration of 185 U/mL for 2 h, at 37°C and moderate shaking. The endonuclease treatment was followed by a filtration step using Sartopure^®^ PP3 filter elements (Sartorius Stedim Biotech GmbH, Germany) with a pore size of 3 µm. The purification process for the VLPs was performed by loading 28 column volumes (CV) (501 mL) of the endonuclease‐treated and filtered cell culture supernatant onto a XK 16/20 column packed with 17.9 mL of Fractogel^®^ EMD TMAE Hicap (M) resin referred in the text as Fractogel^®^‐TMAE (Merck). Buffer A consisted of 50 mM HEPES (pH 7.2) and buffer B of 50 mM HEPES, 2 M NaCl (pH 7.2). A flow rate of 3.6 mL/min was used throughout the whole purification run to ensure a residence time of 5 min. In order to have the same conductivity as in the loading material, the column was equilibrated with 5% B for 5 CV. After loading, the column was washed for 6 CV with equilibration buffer (5% B) to remove all unbound material. Column‐bound material was eluted using a salt linear gradient from 5 to 60% B in 25 CV, followed by a regeneration step at 100% B for 4 CV. The column was then sanitized with 0.5 M NaOH for 3 CV. Flow‐through (FT) fractions were collected with a volume of 100.2 mL (in total five fractions). Elution fractions were collected in 1.6 mL fractions in 96‐deep well plates, further analysed by at‐line HPLC‐multiangle light scattering (MALS) [28] and then pooled according to the chromatogram and stored at 4°C until further use.

### 2.5 Determination of total protein content and DNA content

Total protein and dsDNA quantification were determined as previously described in Ref. [6]. Briefly, for quantification of the total protein the Bradford Assay was used in 96‐well microplate format according to the manufacturer's instructions. Calibration curves were obtained by diluting BSA standard with TE‐buffer to concentrations ranging from 25 to 200 µg/mL. dsDNA was determined by Quant‐iT™ PicoGreen^®^ dsDNA kit (Life Technologies, Waltham, MA, USA) in 96‐well microplate format according to the manufacturer's instructions. Signals for protein (595 nm) and dsDNA content (*λ*_excitation_ = 480 nm, *λ*_emission_  =  520 nm) were measured by Tecan Infinite^®^ 200 Pro (Tecan, Männedorf, Switzerland).

### 2.6 Sodium dodecyl sulphate polyacrylamide gel electrophoresis and Western blot

SDS‐PAGE was performed as previously described in Ref. [6]. Protein bands were stained using silver staining. All solutions used and a full protocol for the silver stain are described in the Supporting Information A, Protocol for Silver Stain.

For Western blot analysis, proteins were blotted as already described in Ref. [6]. For detection of the VLP's capsid protein, a primary antibody against HIV‐1 p24 (ab9071, Abcam) was used. For the detection of the VLP's membrane protein, a primary antibody against H1 influenza A virus H1N1 HA (GTX127357, GeneTex) was used. The primary antibodies were diluted 1:1000 in phosphate buffered saline (PBS)‐T containing 1% BSA (incubation buffer). Anti‐mouse IgG (γ‐chain specific)‐alkaline phosphatase antibody (#3438, Sigma Aldrich) diluted 1:1000 in incubation buffer was used as secondary antibody. For the detection of the baculovirus capsid protein a primary antibody against vp39 was used. For the detection of the membrane glycoprotein gp64 a primary antibody against ACV5 was used (*ab49581*, Abcam, London, UK). The membranes were incubated for 2 h with the corresponding primary antibodies (vp39 diluted 1:50 and ACV5 diluted 1:5000 in incubation buffer). After washing, membranes were incubated for 1 h with the secondary antibody (anti‐mouse IgG (γ‐chain specific)‐alkaline phosphatase antibody), diluted 1:1000 in incubation buffer. For visualization, the membranes were incubated in 10 mL premixed BCIP^®^/NBT solution (Sigma Aldrich) for 2–3 min. Results were evaluated by visual estimation.

### 2.7 Nanoparticle tracking analysis

The determination of the particle concentration and particle size distribution by nanoparticle tracking analysis (NTA) was performed as described in Ref. [6], using a NanoSight NS300 (Malvern Panalytical Ltd., Worcestershire, UK) with a blue laser module (488 nm) and a neutral density filter. Samples were diluted in particle‐free water in order to obtain 20–100 particles per frame. In total, three different dilutions were measured per sample. All measurements were performed at 25°C and videos of 30 s were captured. All particles and a selected particle size range, especially for detection of VLPs between 100 and 200 nm, were considered for sample evaluation. Capture settings (camera level and focus) were adjusted manually, prior to the measurements. For determination of particle concentration, each dilution was measured five times. In total, 15 videos were analyzed for each sample with the NTA 2.3 software.

### 2.8 Tissue culture infective dose 50 on *Spodoptera frugiperda* 9 cells

Quantification of infectious baculovirus titer was performed with tissue culture infective dose 50 (TCID50) on *Spodoptera frugiperda* 9 (Sf9) cells. Sf9 cells in exponential phase were diluted to 0.4 × 10^6^ cells/mL, 100 µL of this dilution was dispensed into each well of a 96‐well plate and incubated for at least 1 h at 27°C to allow cell attachment. Each sample was done in duplicates. Samples were prediluted with HyClone medium (Hyclone SFM5Insect, GE Healthcare) 1:10, in the plates 1:5 dilutions were performed. Virus dilutions were transferred to the 96‐well plates with the attached Sf9 cells. A volume of 30 µL of each virus dilution was added to each well. Plates were incubated at 27°C for at least 7 days. After incubation, the plates were inspected under the Leica DM IL LED Inverted Laboratory Fluorescence Microscope (Leica Microsystems, Wetzlar, Germany). Each well with any sign of infection was counted as a positive well.

### 2.9 Transmission electron microscopy

TEM was used for particle visualization, especially to analyze the presence, integrity, and morphology of particles present across the entire purification run. Sample preparation was performed by negative staining with 1% uranyl acetate as described in Ref. [6]. Images were taken using a Tecnai G2 200 kV transmission electron microscope (FEI, Eindhoven, The Netherlands).

### 2.10 Protein identification and peptide analysis using LC–ESI‐MS

Protein identification and peptide analysis were done as already described in Ref. [6]. The files were searched against the SwissProt database (https://www.ebi.ac.uk/uniprot) against *Trichopulsia* ni (taxonomy ID: 7111) and with special focus on proteins for detection of *Autographa california nuclear polyhedrosis* (AcNPV, taxonomy ID: 46015), HIV‐1 (taxonomy ID: 11676, strain: HIV‐1 HXB2), and Influenza A virus (taxonomy ID: 11320, strain: A/Puerto Rico/8/1934).

### 2.11 HPLC‐multiangle light scattering

At‐line MALS measurements for the determination of the LS intensity were performed using an Ultimate 3000 HPLC system (Thermo Fisher) with a quaternary LPG‐3400SD pump, a WPS‐3000TSL autosampler, and a DAD 3000 UV‐detector. Mobile phase consisted of 50 mM HEPES, 100 mM NaCl, pH 7.2. A sample volume of 50 µL was injected in bypass mode using a flow rate of 0.3 mL/min. All samples were measured in duplicates. MALS signals were acquired by the DAWN HELEOS 18‐angle detector (Wyatt, Santa Barbara, CA, USA). For HPLC programming, Chromeleon 7 software (Thermo Fisher) was used. MALS data collection and analysis were performed with ASTRA software, version 6.1.2 (Wyatt).

### 2.12 HPLC–SEC coupled with multiangle light scattering

Relevant samples were analyzed by HPLC‐SEC‐MALS in order to determine particle composition and estimate purity. All experiments were performed using the HPLC system mentioned in 2.11 with the Chromeleon 7 software (Thermo Fisher Scientific) for method programming, control and data acquisition. A TSKgel G5000PWXL column (300.0 × 7.8 mm i.d.) combined with a TSKgel PWXL guard column (40.0  × 6.0 mm i.d.) (Tosoh Bioscience, Stuttgart, Germany) was used. A volume of 50 µL of each sample was injected. The flow rate was 0.3 mL/min. Isocratic elution was performed with 50 mM HEPES, 100 mM NaCl, pH 7.2. UV signals at 280 and 260 nm were recorded by the Chromeleon software and LS signal was acquired with a DAWN HELEOS 18‐angle detector (Wyatt) with the Astra Software 5.3.4 (Wyatt). Data evaluation was performed in Astra 6.1.2.

## 3 RESULTS AND DISCUSSION

A downstream process based on strong AIEX using Fractogel^®^‐EMD TMAE Hicap (M) as stationary phase was developed for capturing and separating enveloped HIV‐1 gag H1 VLPs from baculovirus. The results of the VLP capture and purification are described in Section 3.1 and the purity and particle content of the main particle containing fractions are compared in Section 3.2.

### 3.1 Purification of virus‐like particles using Fractogel^®^‐TMAE

**FIGURE 1:**
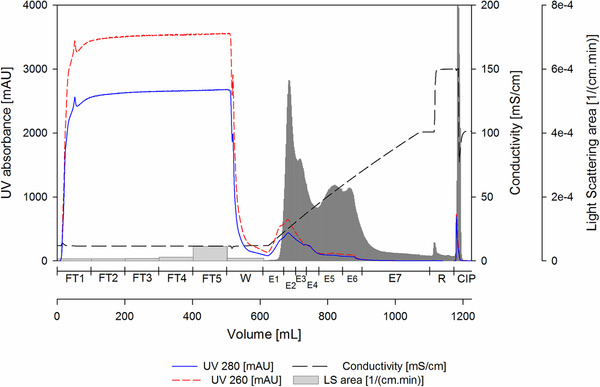
Chromatographic purification of HIV‐1 gag H1 VLPs from baculovirus, produced in *Tnms*42 insect cells, with Fractogel^®^‐TMAE using a linear gradient elution from 100 to 1000 mM NaCl (Buffer A: 50 mM HEPES, pH 7.2; Buffer B: 50 mM HEPES, 2 M NaCl, pH 7.2). Loading material (28 CV, 501 mL) was endonuclease treated and filtered (3 µm). Grey bars represent the area under the curve of the light scattering intensity (LS) measurements performed on MALS detector. CIP: cleaning in place (0.5 M NaOH), E1–E7: elution fractions 1–7, FT1–FT5: flow‐through fractions 1–5, R: regeneration (100% B)

**FIGURE 2:**
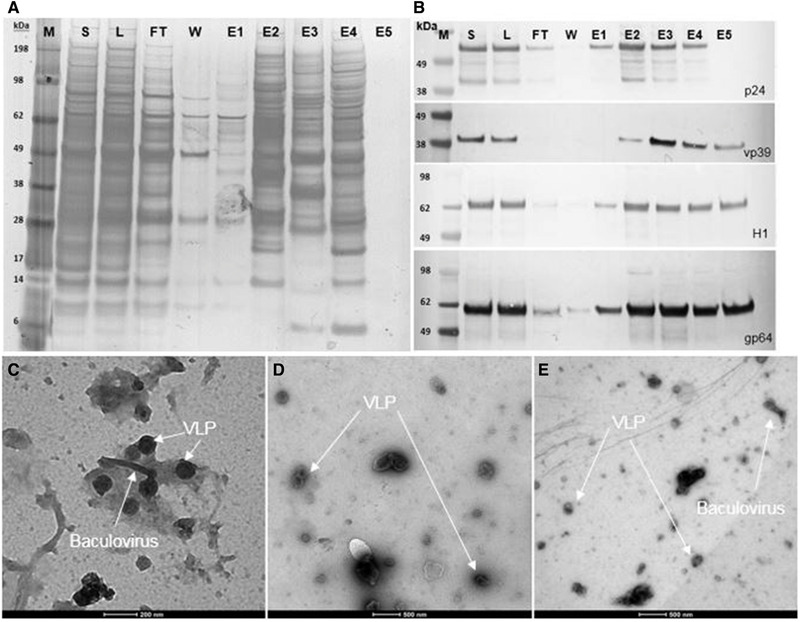
(A) SDS–PAGE, (B) Western blot analysis and of the pooled fractions from the purification run represented in Figure 1. (C) to (E) Electron microscopy micrographs of loading material (L) and main elution fractions E2 and E3, respectively. E1–E5: elution fractions 1–5, FT: pooled flow‐through, L: loading material (endonuclease treated and filtered), M: molecular weight marker

**FIGURE 3:**
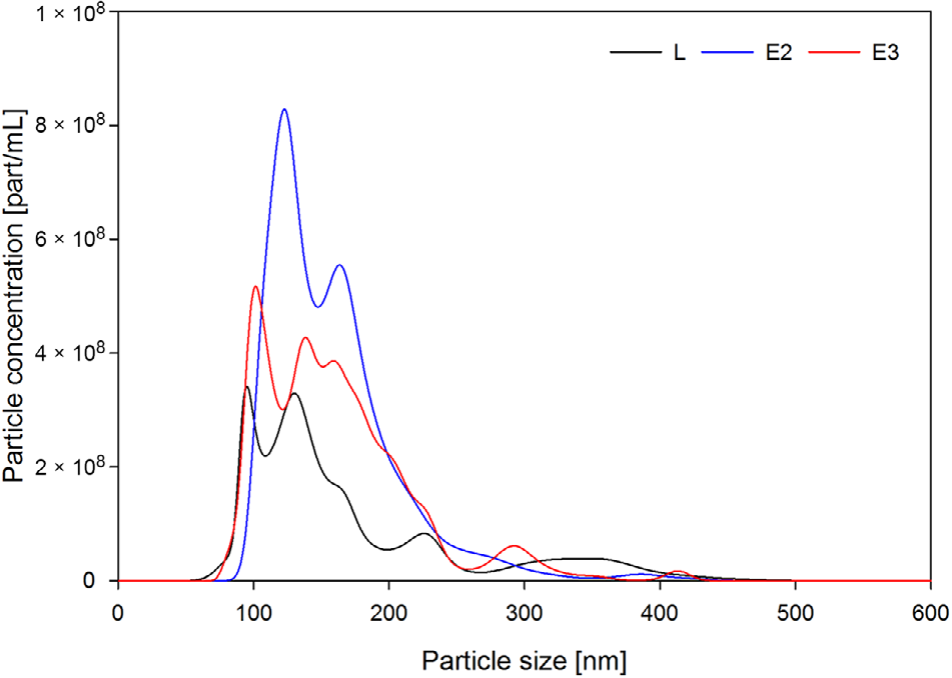
Particle size distribution measured by nanoparticle tracking analysis of loading material (L) and the main particle containing fractions E2 (VLP containing fraction) and E3 (VLP and coelution of baculovirus)

**FIGURE 4:**
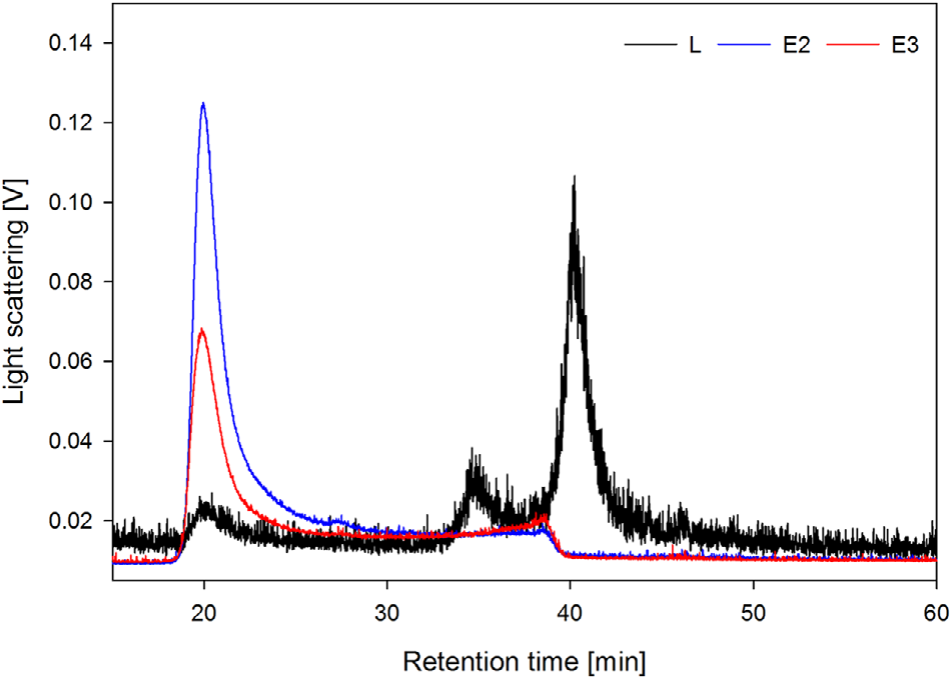
Analysis of the loading material (L) and the main elution fractions E2 and E3 from the purification run represented in Figure 1 by analytical size exclusion chromatography coupled to MALS

To estimate the dynamic binding capacity, a 1 mL Fractogel^®^‐TMAE prepacked MiniChrom column 8  ×  20 mm (Merck) was overloaded with clarified and endonuclease pretreated *Tnms*42 cell culture supernatant. LS signal of the FT fractions during the loading phase shows particle breakthrough after approximately 30 mL, equivalent to 30 CV (Supporting Information B, Figure S1, Fraction FT1). In order to avoid product loss due to overloading, the 17.9 mL Fractogel^®^‐TMAE column was loaded with 28 CV. Accordingly, 501.0 mL of clarified, endonuclease‐pretreated and filtered *Tnms*42 supernatant was directly loaded onto the column. A salt linear gradient from 100 to 1000 mM NaCl over 25 CV allowed the elution of bound particles from the column (Figure 1). Collected fractions were analyzed by at‐line MALS [6]. Sample pooling was performed considering both, UV absorbance and LS area [8, 28, 29]. For the pooled samples, dsDNA was determined by Picogreen Assay (Table 1 and Supporting Information B, Table S1), total protein was quantified by Bradford Assay (Table 1 and Supporting Information B, Table S1) and specific protein contents were accessed by SDS–PAGE (Figure 2A), Western Blots (Figure 2B), and MS (Supporting Information C). Particle content (Table 1 and Supporting Information B, Table S1) and particle size distribution (Figure 3) were measured by NTA and quantification of infectious baculovirus titer by TCID50 (Supporting Information B, Table S2). The combination of these data allowed the evaluation of the process performance in terms of recovery and purity. Yield cannot be calculated in a direct manner, because there is no method to specifically quantify the VLPs in the crude material in presence of baculovirus and many process related impurities. Additionally, biochemical markers targeting specific proteins are not VLP specific once they would also measure free protein in solution that did not assemble into VLPs. Treatment of the supernatant with endonuclease allowed a depletion of 57% dsDNA from 1583.6 ng/mL in the supernatant to 683.8 ng/mL in the loading material (Table 1). For specific detection of VLPs and baculovirus, we used Western blot analysis against the proteins HIV‐1 p24 (band at 55 kDa) and baculovirus vp39 (band at 39 kDa), respectively, because these are the main capsid proteins. Additionally, influenza A virus H1N1 HA (band at 64 kDa) and the baculovirus major envelope glycoprotein gp64 (band at 59 kDa) were used to detect membrane proteins for VLP and baculovirus, respectively. However, membrane proteins were identified in all particle containing fractions (Figure 2B), which was expected once the different particles share the same budding mechanism at the cell membrane. Particle concentration measured by NTA reveals that E2 and E3 contain the majority of the eluted particles (Table 1). These results were supported by the HPLC‐SEC‐MALS measurements, in which particles elute in the void volume of the column after a retention time of 20 min and the highest LS signals can be observed for E2 and E3 (Figure 4). Considering the LS data, particles were also concentrated from the loading material to E2 and E3 (Figure 4). The UV280 data of the analytical SEC measurements were evaluated in order to infer about the purity level of the main elution fractions E2 and E3 (Supporting Information B, Figure S2). Moreover, when looking at the UV280 data in E2 and E3, the reduced signal indicates reduction in impurity content. Also, a different protein pattern between E2 and E3 can be observed on the SDS‐PAGE (Figure 2A), indicating the elution of different particle populations. On the SDS‐PAGE in lane E3, a very dense band at 39 kDa (Figure 2A) which is not visible in E2, suggests the elution of baculovirus. Considering the Western blot results against HIV‐1 p24 and baculovirus vp39, in E2 a dense band against the capsid protein p24 is visible while only a faint band for vp39 is present (Figure 2B). This indicates the enrichment of VLPs and separation from baculovirus in E2. Contrariwise, in E3 the vp39 band is denser indicating the elution of baculovirus. VLP enrichment in E2 and starting coelution of baculovirus can also be confirmed by TEM pictures (Figure 2C to E). Since E2 and E3 are not resolved peaks (Figure 1), the separation of VLPs and baculovirus could be improved by either using a narrower pooling criteria or by optimizing the elution gradient. Elution of VLPs in E2 is further supported by NTA results (Table 1), which showed that E2 contained 20% of the loaded particles (100–200 nm). Particle size distribution showed that the particles in E2 had a mean diameter of 158.4 nm, the typical diameter of VLPs based on the HIV‐1 gag construct [8] (Figure 3). Additionally, 81% of the particles in E2 have a diameter between 100 and 200 nm (NTA), while in E3 particles have a slightly wider particle size distribution (Figure 3) with mean size of 161.3 nm, which can be explained by the coelution of baculovirus. This supports the findings of the Western blot analysis (Figure 2B). Proteomic analysis was performed by LC‐ESI‐MS in order to identify specific proteins in the main particle containing fractions. Considering E2 and E3, in total 145 and 161 proteins were identified against the host *Trichopulsia ni* database, respectively (Supporting Information C). Additionally, a search against the specific strains used for HIV‐1, influenza and baculovirus was performed. As shown by the Western blot analysis, both membrane proteins (H1 and gp64) were detected in both samples (E2 and E3). In E2, the capsid protein HIV‐1 gag (specific for VLPs) was identified again confirming the Western blot results. Additionally, six different proteins from baculovirus (AcMNPV) were present in E2, indicating coelution, however, since the peaks are not fully resolved and MS is a very sensitive detection method this is expected. In E3, 15 proteins specific for baculovirus were identified, including the major capsid protein vp39, which was not detected in E2 (Supporting Information C). An additional purification run with *Tnms*42 cell culture supernatant previously stored at −80°C was performed and showed the same elution profile as the purification run performed using fresh material (Supporting Information, Figure S3). First, a pure fraction of VLPs is eluting in E2.1 and in E3, baculovirus starts to coelute, which can be confirmed by Western blot analysis performed against the specific capsid proteins p24 and vp39 (Supporting Information B, Figure S4). After thawing, the loading material infectivity regarding baculovirus was 3.0 × 10^6^ TCID50/mL (Supporting Information B, Table S2). After purification, a virus clearance of log 4.3 and 3.2 was achieved for E2.1 (main VLP fraction) and E3 (VLP‐baculovirus coelution), respectively. Considering the particle size distribution, Western blot profiles, proteomic data, TEM pictures and TCID50 values of the main particle containing fractions, we conclude that a HIV‐1 gag H1 VLPs enriched fraction elutes in E2, and baculovirus coelution starts in E3.

**TABLE 1:** Mass balance of the purification run for HIV‐1 gag H1 VLPs on a 17.9 mL Fractogel^®^‐TMAE column by linear gradient elution

Sample	volume [mL]	Particles (1–1000 nm) [part/mL]	Recovery [%]	Particles (100–200 nm) [part/mL]	Recovery [%]	Total protein [μg/mL]	dsDNA [ng/mL]]
S	501.0	‐	‐	‐	‐	249.5	1583.6
L	501.0	2.6 × 10^10^	100	1.8 × 10^10^	100	221.6	683.8
E2	35.2	6.0 × 10^10^	16	4.9 × 10^10^	20	204.9	204.2
E3	32.0	4.0 × 10^10^	10	2.9 × 10^10^	10	252.0	173.1

Loading material was endonuclease treated and 3 µm filtered *Tnms*42 cell culture supernatant. E2–E3: elution fractions 2–3 (main particle containing fractions), L: loading material, S: *Tnms*42 cell culture supernatant.

### 3.2 Purity of virus‐like particles

**FIGURE 5:**
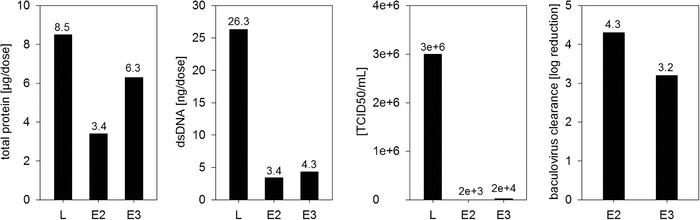
Purity of the loading material (L) and the main particle elution fractions E2 and E3 from the Fractogel^®^‐TMAE purification run calculated based on μg protein and ng dsDNA/dose and baculovirus clearance based on TCID50/mL and log reduction

Total protein, dsDNA, and particle contents of E2 and E3 were determined and normalized per vaccine dose (10^9^ particles) in order to allow the comparison of the main particle fractions regarding its purity. The total protein content was 3.4 µg per dose for E2 and 6.3 µg per dose for E3 (Figure 5). The dsDNA content was similar for both fractions (3.4 ng/dose for E2 and 4.3 ng/dose for E3, Figure 5) and already meet the requirements of the regulatory agencies with <10 ng of residual dsDNA per dose [30]. Performance of the purification run was calculated based on the number of vaccination doses per liter loading material. We were able to purify 4200 vaccination doses per liter pretreated *Tnms*42 cell culture supernatant using a 17.9 mL Fractogel^®^‐TMAE column.

## 4 CONCLUDING REMARKS

In our work, we demonstrate that polymer‐grafted anion exchangers are capable of efficiently capturing chimeric HIV‐1 gag influenza H1 VLPs directly from clarified and endonuclease‐treated insect cell culture supernatant. Moreover, this method allowed the separation of the VLPs from process related impurities, such as host cell proteins and dsDNA, and most importantly from baculovirus, in a single step. A reduction of 94% total protein and 98% dsDNA was achieved for the main product fraction. When considering 10^9^ particles as a vaccination dose, purified influenza VLPs already meet the requirements of the regulatory agencies with <10 ng residual of dsDNA. From each liter of pretreated cell culture supernatant, we were able to process 4200 vaccination doses with Fractogel^®^‐TMAE. The process is simple with a very low number of handling steps and has the characteristics to become a platform for purification of these types of VLPs.

## CONFLICT OF INTEREST

The authors have declared no conflict of interest.

## Supporting information

Supporting information

Supporting information
