# Supplementary material for: Separation of influenza virus‐like particles from baculovirus by polymer‐grafted anion exchanger
Source: J Sep Sci. 2020 Apr 30;43(12):2270–8. doi: 10.1002/jssc.201901215 (PMC7318652; doi:10.1002/jssc.201901215)
Supplement: Supplementary file 1 — Supporting information [file JSSC-43-2270-s002.doc]

## Supplementary Material A

**Separation of influenza virus-like particles from baculovirus by polymer grafted ion-exchangers**

Katrin Reiter^1^, Patricia Pereira Aguilar^1,2^, Dominik Grammelhofer^1^, Judith Joseph^1^, Petra Steppert^2^, Alois Jungbauer^1,2*^

^1^ Austrian Centre of Industrial Biotechnology, Vienna, Austria

^2^ Department of Biotechnology, University of Natural Resources and Life Sciences, Vienna, Austria

*Corresponding author

Univ.Prof. Dipl.-Ing. Dr.nat.techn. Alois Jungbauer

Department of Biotechnology

University of Natural Resources and Life Sciences Vienna,

Muthgasse 18, 1190 Vienna

Phone: +43 1 47654-79083

Fax: +43 1 47654-79009

E-mail: [alois.jungbauer@boku.ac.at](mailto:alois.jungbauer@boku.ac.at)

**Supplemenatary Material A, Protocol for Silver Stain**

**Solutions**

Fixing Solution 30 min +

| Ethanol (96%) | 500 mL |
| --- | --- |
| Acetic Acid (100%) | 100 mL |
| RO-Water | Fill to 1 L |

Incubation Solution 30 min +

|  | **For 1 L** | **One gel (50 mL)** |
| --- | --- | --- |
| Ethanol (96%) | 300 mL | 15 mL |
| Na-acetate (waterfree) | 68 g | 3.4 g |
| Thiosulfate-pentahydrate  (Na_2_S_2_O_3_.5H_2_O) | 2 g | 0.1 g |
| RO-Water | Fill to 1 L | Fill to 50 mL |
| **Add before use:** | | |
| Glutaraldehyde (50% in water (5.6 M) | 2.5 mL | 125 µL |

**wash 3x5 min**

**Silver Solution 20 min**

|  | **For 1 L** | **One gel (50 mL)** |
| --- | --- | --- |
| Silver nitrate (AgNO_3_) | 1 g | 50 mg |
| RO-Water | Fill to 1 L | Fill to 50 mL |
| **Add before use:** | | |
| Formaldehyde 35% | 200 µL | 10 µL |

**wash 2-3x**

Developing Solution ~ 5 min

|  | For 1 L | One gel (150 mL) |
| --- | --- | --- |
| Sodium carbonate (Na_2_CO_3_) | 25 g | 3.75 g |
| RO-Water | Fill to 1 L | Fill to 150 mL |
| **Add before use:** | | |
| Formaldehyde 35% | 100 µL | 15 µL |

Stop Solution 10 min

| EDTA/Triplex | 14.6 g |
| --- | --- |
| RO-Water | Fill to 1 L |

**Silver Staining**

- All steps have to be performed on a lab shaker, the fluid should cover the gel (~30 mL)
- Put the gel into an appropriate container and add 50 mL Fixing solution for at least 30 minutes if the Gel isn’t already fixed
- The **Glutaraldehyd/Formaldehyd** is added to 50 mL of the Solution in a Greiner tube right before use
- Incubate it **30 minutes** in 50 mL **Incubation Solution**
- Wash **3 times for 5 minutes** in RO-Water
- Incubate **20 minutes** in 50 mL **Silver Solution**
- **Rinse shortly 2-3 times** with water to remove the Silver Solution
- Incubate in 50 mL **Developing Solution** for approximately ~ **5 minutes** until the protein bands are clearly visible. Change the Developing Solution every 2-3 minutes to get better results.
- Incubate the gel min. **10 min** in 50 mL **Stop Solution** to stop the reaction. (gel can be kept in stop solution until scanning)
- Scan the gel and store the data.

## Supplementary Material B

**Separation of influenza virus-like particles from baculovirus by polymer grafted ion-exchangers**

Katrin Reiter^1^, Patricia Pereira Aguilar^1,2^, Dominik Grammelhofer^1^, Judith Joseph^1^, Petra Steppert^2^, Alois Jungbauer^1,2*^

^1^ Austrian Centre of Industrial Biotechnology, Vienna, Austria

^2^ Department of Biotechnology, University of Natural Resources and Life Sciences, Vienna, Austria

*Corresponding author

Univ.Prof. Dipl.-Ing. Dr.nat.techn. Alois Jungbauer

Department of Biotechnology

University of Natural Resources and Life Sciences Vienna,

Muthgasse 18, 1190 Vienna

Phone: +43 1 47654-79083

Fax: +43 1 47654-79009

E-mail: alois.jungbauer@boku.ac.at

Figure S1


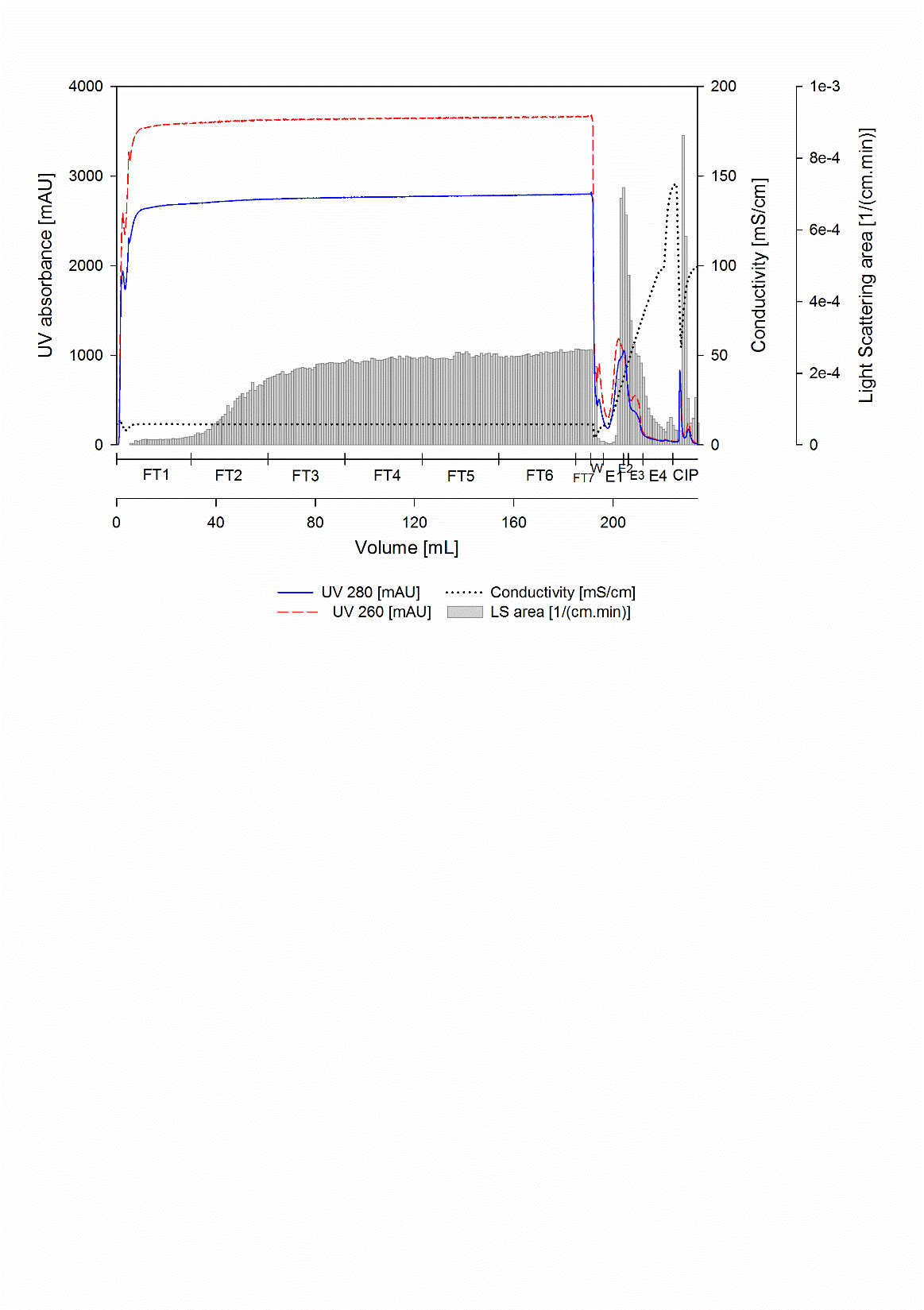


Figure S1: Chromatographic purification of HIV-1 gag H1 VLPs from baculovirus produced in *Tnms*42 insect cells with a 1 mL Fractogel®-TMAE prepacked MiniChrom column 8 × 20 mm (Merck KGaA, Darmstadt, Germany), using a linear gradient elution from 100-1000 mM NaCl (Buffer A: 50 mM HEPES, pH 7.2; Buffer B: 50 mM HEPES, 2 M NaCl, pH 7.2). Column was overloaded with 191 CV. The loading material was endonuclease treated and filtered (3 µm). Grey bars represent the area under the curve of the light scattering intensity (LS) measurements performed on MALS detector. FT1-FT7: flow-through fractions 1-7, E1-E4: elution fractions 1-4, CIP: cleaning in place (0.5 M NaOH).

Figure S2


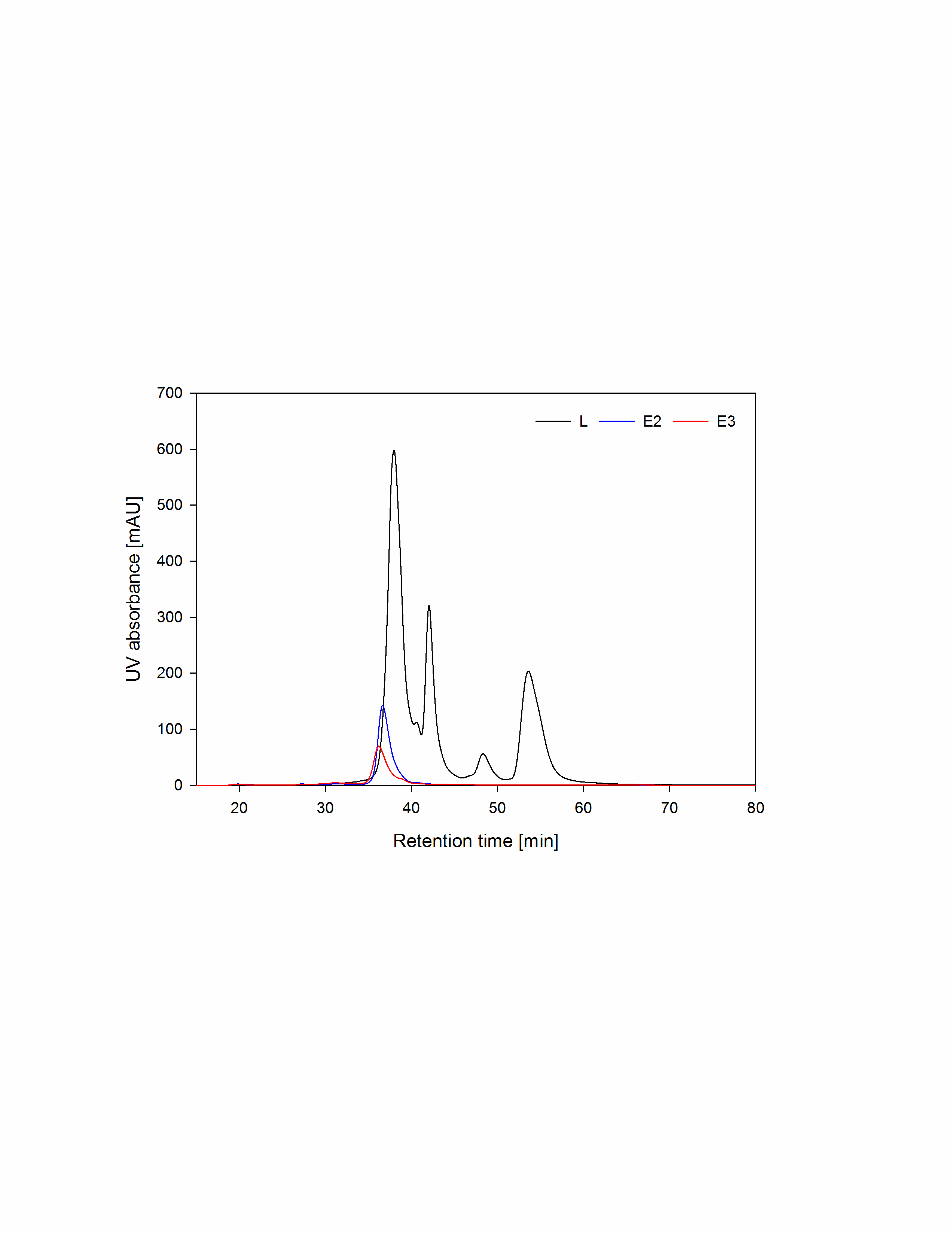


Figure S2: UV280 absorbances for analysis of the loading material (L) and the main elution fractions E2 and E3 from the purification run represented in Figure 1 by HPLC-SEC.

Figure S2


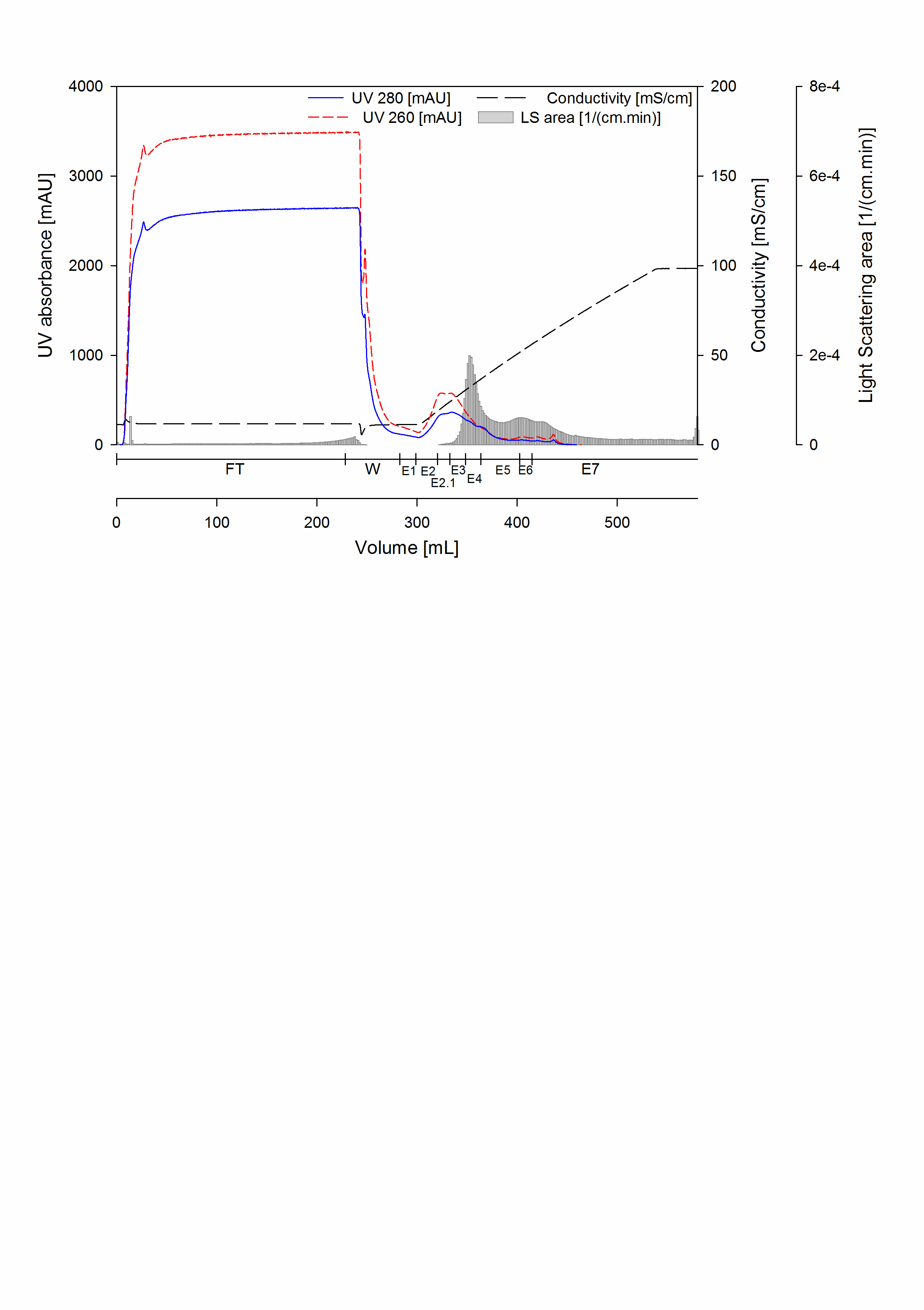


Figure S2: Chromatographic purification of HIV-1 gag H1 VLPs from baculovirus produced in *Tnms*42 insect cells on a 9.5 mL Fractogel®-TMAE column, using a linear gradient elution from 100-1000 mM NaCl (Buffer A: 50 mM HEPES, pH 7.2; Buffer B: 50 mM HEPES, 2 M NaCl, pH 7.2). Column was loaded with 25 CV. The loading material previously stored at -80°C, was thawed, endonuclease treated and filtered (3 µm). Grey bars represent the area under the curve of the light scattering intensity (LS) measurements performed on MALS detector. FT: flow-throug, E1-E7: elution fractions 1-7

Figure S3


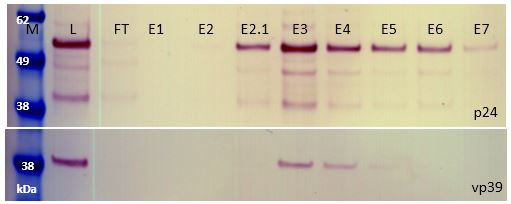


Figure S3: Characterization of the pooled fractions from the Fractogel®-TMAE repetition run using *Tnms*42 cell culture supernatant represented in Figure S3. Western blots against the specific capsid proteins HIV-1 p24 and vp39 for VLP and baculovirus detection, respectively. M: molecular weight marker, L: loading material (c-LEcta Denarase® treated and filtered), FT: flow-through, E1-E7: elution fractions 1-7.

Table S1: Total mass balance of the purification run using Fractogel®-TMAE for the separation of HIV-1 gag H1 VLPs and baculovirus, using a 17.9 mL packed column. S: Tnms42 cell culture supernatant containing HIV-1 gag H1 VLPs and BV, L: loading material (endonuclease treated and filtered); FT1-5: flow-through fractions 1-5; W: wash; E1-E7: elution fractions 1-7, R: regeneration (100% B), CIP: cleaning-in-place (0.5 M NaOH).

| **sample** | **volume**  **[mL]** | **particles**  **(1-1000nm)**  **[part/mL]** | ***r*ecovery**  **[%]** | **particles**  **(100-200nm)**  **[part/mL]** | **recovery**  **[%]** | **total protein**  **[µg/mL]** | **dsDNA**  **[ng/mL]** |
| --- | --- | --- | --- | --- | --- | --- | --- |
| **S** | 501 | - | - | - | - | 249.5 | 1583.6 |
| **L** | 501 | 2.60E+10 | 100% | 1.80E+10 | 100% | 221.6 | 683.8 |
| **FT1** | 100.2 | 1.30E+09 | 1% | 9.40E+08 | 1% | 77.6 | 199.6 |
| **FT2** | 100.2 | 1.30E+09 | 1% | 9.40E+08 | 1% | 119.8 | 325.6 |
| **FT3** | 100.2 | 1.60E+09 | 1% | 1.20E+09 | 1% | 99.2 | 345.6 |
| **FT4** | 100.2 | 1.20E+09 | 1% | 9.30E+08 | 1% | 114.8 | 338.4 |
| **FT5** | 100.2 | 3.90E+08 | 0% | 9.70E+07 | 0% | 123.2 | 369.5 |
| **W** | 107.4 | 5.90E+08 | 0% | 3.54E+09 | 0% | < LLOQ | 87.4 |
| **E1** | 60.8 | 1.50E+09 | 1% | 1.30E+09 | 1% | 55 | 41.5 |
| **E2** | 35.2 | 6.00E+10 | 16% | 4.90E+10 | 19% | 204.9 | 204.2 |
| **E3** | 32 | 4.00E+10 | 10% | 2.90E+10 | 10% | 252 | 173.1 |
| **E4** | 36.8 | 1.40E+10 | 4% | 9.30E+09 | 4% | 158.1 | 115 |
| **E5** | 70.4 | 1.30E+10 | 7% | 8.50E+09 | 7% | 192.7 | 460.5 |
| **E6** | 57.6 | 5.90E+09 | 3% | 3.60E+09 | 2% | 210.1 | 837.5 |
| **E7** | 199.5 | 5.00E+08 | 1% | 2.90E+08 | 1% | < LLOQ | 39.1 |
| **R** | 71.6 | n.d. | n.d. | n.d. | n.d. | < LLOQ | < LLOQ |
| **CIP** | 52.8 | n.d. | n.d. | n.d. | n.d. | 81.2 | < LLOQ |
| **Sum** |  |  | **45%** |  | **48%** |  |  |

n.d.: not determined

< LLOQ: under the lowest limit of quantification

Table S2: Characterization of the pooled fractions from the Fractogel®-TMAE repetition represented in Figure S3. L: loading material (endonuclease treated and fitlered), E2.1 and E3: main elution fractions

| **sample** | **volume**  **[mL]** | **virus infectivity**  **[TCID50/mL]** | **log**  **reduction** |
| --- | --- | --- | --- |
| **L** | 236.3 | 3.0E+06 | - |
| **E2.1** | 12.8 | 1.9E+03 | 4.3 |
| **E3** | 16.0 | 1.9E+04 | 3.2 |
